# Avoiding allogenic blood transfusions in endoscopic angiofibroma surgery

**DOI:** 10.1186/s40463-016-0135-5

**Published:** 2016-04-11

**Authors:** Hisham Wasl, Jessica McGuire, Darlene Lubbe

**Affiliations:** Division of Otolaryngology, University of Cape Town, Cape Town, South Africa

## Abstract

**Background:**

Surgical approaches for many tumours are often limited by blood loss, exposure and risk to vital anatomical structures. Therefore, the standard of care for certain skull base tumours has become endoscopic transnasal resection. Other surgical disciplines often use cell salvage techniques, but review of the otolaryngology literature revealed very few case reports. This study investigated the value and safety of salvage-type autologous blood transfusion during the endoscopic resection of juvenile nasopharyngeal angiofibromas (JNA).

**Methods:**

JNA is a rare vascular nasal tumour and the study extended over a 3-year period to obtain adequate patient numbers. All patients undergoing endoscopic resection during this period were included in the population sample. Ten patients with JNA were identified and underwent embolization prior to the endoscopic resection. In all cases the intraoperative blood salvage apparatus was used. Close post-operative monitoring was performed.

**Results:**

Homologous blood transfusion could be avoided in all cases. Postoperative monitoring revealed transient bacteraemia in two cases where the leukocyte filter was not used, but no evidence of septicaemia.

**Conclusions:**

Perioperative cell saver and autologous blood transfusion in endonasal JNA surgery is safe. Homologous blood transfusion can be avoided by using this technique. The use of cell salvage allows for single stage surgery without the need to abandon surgery due to excessive blood loss and its future use is promising.

## Background

For more than 25 years, trauma, orthopaedic, urological, liver transplant and cardiac surgery have used intraoperative cell salvage (ICS) techniques [1]. However, in contaminated surgical fields the role of cell salvage techniques has been contentious. Contaminated surgical fields have been previously considered as a relative or absolute contraindication for intraoperative cell salvage. However, it is increasingly being used during trauma surgery even though the surgical field is often deemed contaminated and has been found to be a safe technique. A review of the literature reveals no research about the utilisation of cell salvage techniques in endonasal surgery.

Haemorrhage during tumour surgery often results in the need for intraoperative allogeneic blood transfusion. Many reports have focused on allogenic transfusion risks, such as immunomodulation, transfusion related immune-mediated reactions and infection. It may be associated with increased mortality, myocardial infarction and increased risk of tumour recurrence [2]. ICS autologous blood transfusions reduce the need for allogenic blood transfusions.

Other advantages of ICS autologous blood transfusions are reduced burden on blood donation systems, the blood available is proportional to the blood lost during surgery, there is no haemodilution; and it can be used when significant blood loss is anticipated [3]. Salvaged red cells also have a better oxygen delivery profile, and the system is cost-effective.

Pre-operative embolization is associated with less blood loss. A meta-analysis of the clinical features and treatment outcomes were unable to draw definitive conclusions regarding expected blood loss because most studies do not stratify blood loss according to stage and most studies on JNA are non-comparative case series [4]. At our institute almost all patients with Fisch stage 2c and 3 would require a transfusion, even with pre-operative embolization.

**Fig. 1:**
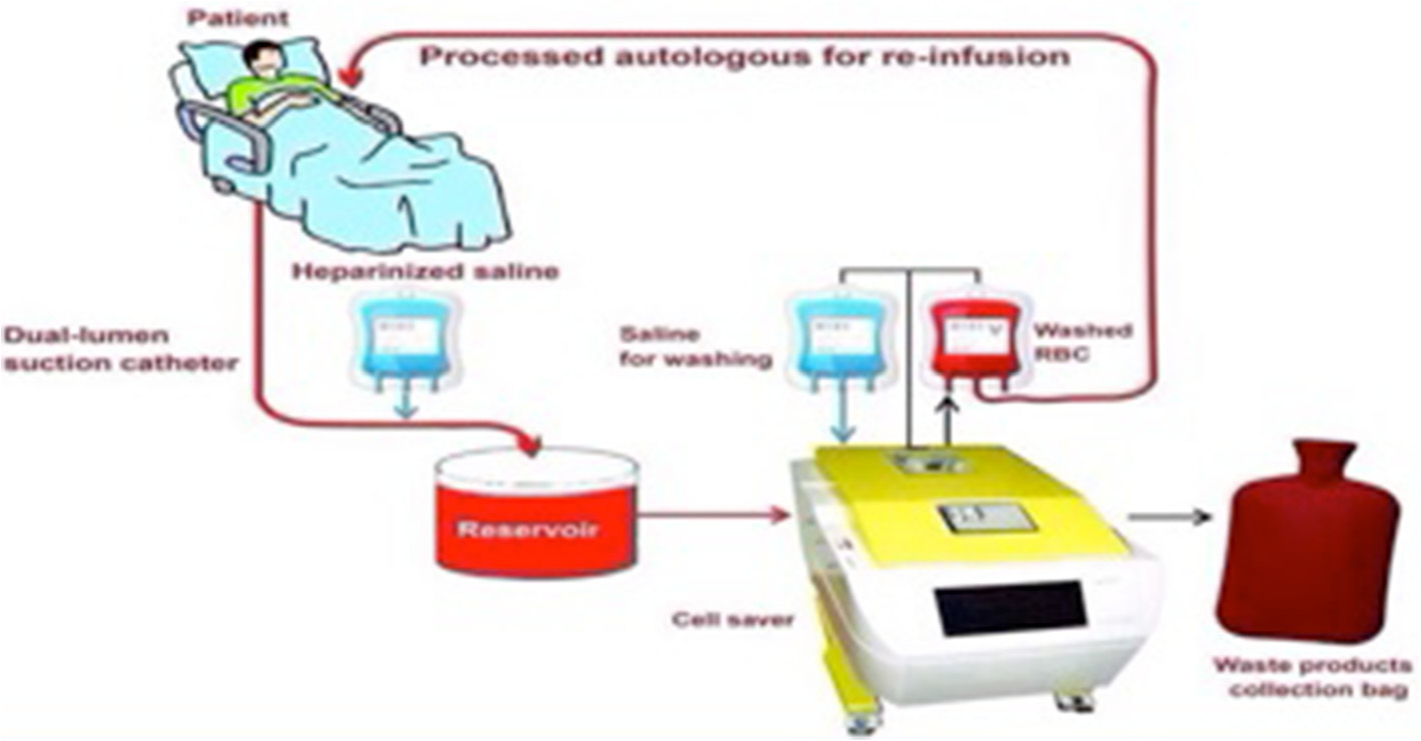
Cell salvage techniques

The autologous red cell recovery system, also known as the “cell saver”, has a double lumen tube that mixes the aspirated blood with a heparin solution and collects it in a reservoir (Fig. 1). A centrifuge is then used to separate erythrocytes from the blood and cell stroma, free haemoglobin and plasma flow to the waste bag. The leukocyte filter is used to provide further protection, and blood is then re-infused into the patient (Fig. 1). The mechanisms of leukocyte reduction by leukocyte depletion filter (LDF) are insufficiently understood. It is widely accepted that centrifugal effects are used to eliminate bacteria with cell saver methods. Bacterial removal mechanisms of the leukocyte depletion filter (LDF) cannot depend on the size of bacteria alone and may be more dependent on adhesive characteristics of the filter, such as the surface structure of the material used in the filter, wettability, surface charge, the disintegration of cells, and deformability of infected cells in the filter [5, 6]. Reports indicate that 97.6 to 100 % of bacterial colony-forming units may be cleared when blood is subjected to a leukocyte filtration system and is washed.

According to our reading, there has been no research done with regards to the role of red cell salvage in endoscopic JNA surgery. This research forms a preliminary study into the role of cell salvage during JNA surgery.

## Methods

We conducted a prospective study over a 3 year period of all patients undergoing endoscopic resection of JNA at 2 centres, by a single surgeon. Ten patients were enrolled, they all had Fisch stage 3 or 4 tumours and all underwent pre-operative embolization, had intra-operative cell salvage and close postoperative monitoring. We received ethics approval from the *Human Research Ethics Committee* of the *University of Cape Town*. Inclusion criteria were an estimated blood loss of more than 500 ml and a patient that may qualify for an intraoperative blood transfusion. No JNA patients were excluded.

Patients were all male and aged between 12 and 20 years. The first patient was a Jehovah’s Witness and refused allogeneic blood transfusion; however the church found autologous blood transfusion via a salvage system acceptable if transfusion was required. No leucocyte filter was available in the government sector hospital and the surgeons advised against surgery since the transfusion likelihood was 100 % for a stage 2c tumour at our institution. However, due to state policy the surgeons were obligated to perform surgery.

**Fig. 2:**
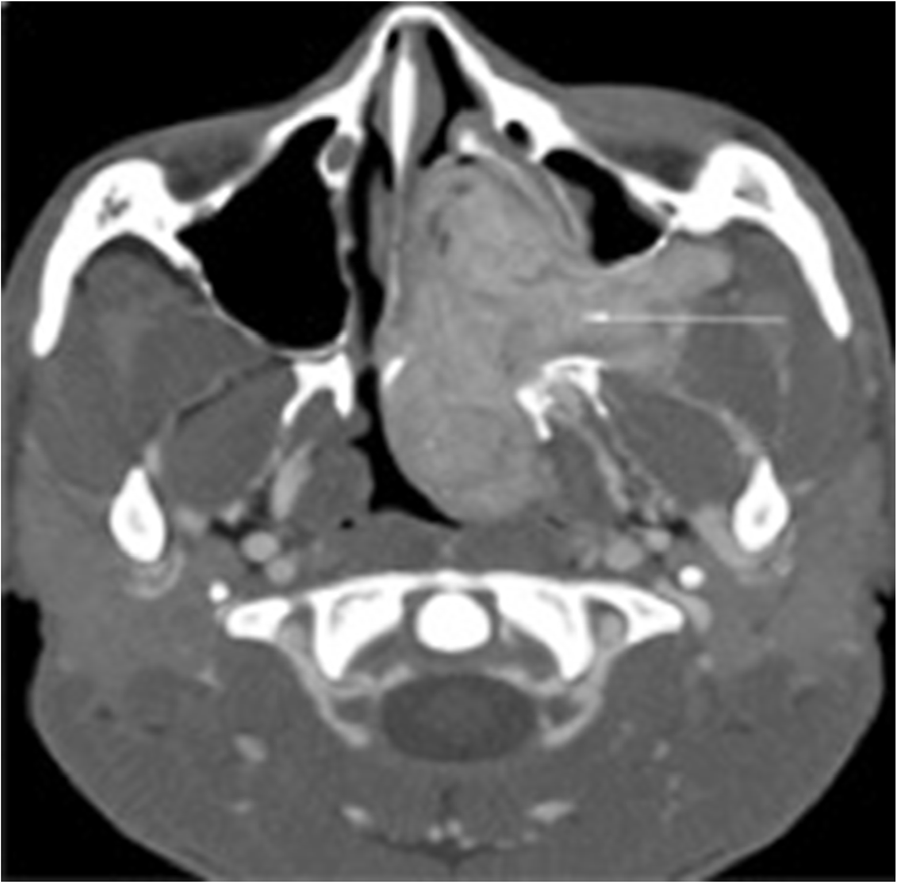
Computed tomography scan showing a Fisch stage 3 JNA

The Fisch staging system for JNA grades tumours invading pterygomaxillary fossa and paranasal sinuses with bony destruction as stage 2. Stage 3 tumours invade the infratemporal fossa, orbit and/or parasellar region, remaining lateral to cavernous sinus (Fig. 2).

Based on the Fisch staging system for JNA, 6 patients had stage 2 tumours and 4 had stage 3 tumours (Table 1). Two patients with stage 3 disease had staged procedures with initial incomplete resection and the rest of the patient had successful complete resection of the tumour at the first surgery. Of the 2 patients requiring a second stage procedure, 1 had unsuccessful preoperative embolization and the first surgery was terminated as a result of bleeding and the other patient had extensive disease and residual tumour which required a second look procedure.

**Table 1:** Summary of results

Patient	Age	Tumour stage Fisch	Intraoperative blood loss (ml)	Cell saver blood transfusion	Nasal swab	Cell saver blood culture	Postoperative blood culture
Patient 1	12	2	500 ml	200 ml	Staphylococcus Aureus	No growth	No growth
Patient 2	19	2	500 ml	300 ml	Staphylococcus Aureus	No growth	No growth
Patient 3	18	3	1700 ml	700 ml	Staphylococcus Aureus	No growth	No growth
Patient 4 No LDF	13	3	2000 ml	1500 ml	Staphylococcus Aureus	Staphylococcus Aureus	Staphylococcus Aureus
Patient 5 Johavah’s Witness	16	3	4000 ml	2500 ml	Streptococcus Viridans	No growth	No growth
Patient 6	14	3	3000 ml	1700 ml	Methicillinresistant Staphylococcus Aureus (MRSA)	No growth	No growth
Patient 7	15	2	1000 ml	600 ml	Staphylococcus Epidermidis	No growth	No growth
Patient 8	13	2	900 ml	400 ml	Streptococcus Viridans	No growth	No growth
Patient 9	16	2	1000 ml	600 ml	Moraxella Catarrhalis	No growth	No growth
Patient 10 No LDF	14	2	700 ml	250 ml	Staphylococcus Aureus	Staphylococcus Aureus	Staphylococcus Aureus

An intra-operative nasal swab was taken to determine the presence of bacteria in the surgical field and to obtain sensitivities in the event that antibiotics were required postoperatively. The cell saver system was used to salvage autologous blood. A second specimen for microscopy, culture and sensitivity (MCS) was taken from the cell saver system to detect the presence of organisms in the autologous blood that was to be transfused back to the patient.

The anaesthetist and surgeon based decisions to transfuse each patient on their haemoglobin and clinical stability. The transfusion trigger was a haemoglobin of 7.0 g/dL or a falling pH level or an increasing anion gap. Blood was collected from the cell saver unit by a technician. Heparin was added to the collected blood, and only processed if a sufficient volume was recovered, or if the patient required a blood transfusion based on the above parameters. The disposables and the leukocyte filter need only be set up when a decision to process the blood is made. We found that using this standby technique could reduce the costs of cell salvage by 90 % if no blood was processed. This is important in resource-restricted hospitals.

The leukocyte filter was not used in two cases, as it was not available in the state hospital at that time. The rationale for proceeding with the autologous blood transfusion was that the patients were covered with broad spectrum antibiotics and the surgical site, although colonised by bacteria, was not infected. United Kingdom guidelines for autologous intraoperative blood transfusion recommend the use of a LDF in surgery for malignant disease, although they state that extensive clinical experience suggests the risk of seeding is not significant. JNA are benign tumours with no risk of distant metastases [7].

Our decision to use the LDF was based on knowledge that the nasal cavity is colonised by bacteria. There are no specific guidelines for the use of LDF in uncontaminated, colonised surgical fields [7].

All patients received prophylactic intraoperative antibiotics and a post-operative course of broad spectrum antibiotics for 48 h. Patients were monitored for signs of infection/ septicaemia by routine methods of temperature and pulse rate. A blood culture was performed 1 day following surgery to exclude a bacteraemia or septicaemia.

Postoperatively, all patients were admitted to the intensive care unit (ICU) for one day for monitoring. Laboratory coagulation tests were taken on admission to ICU, and the international normalised ratio (INR) in the laboratory control group was 1.1.

The mean hospital stay for all patients was 3 days. Nasal packs were removed on the second day after surgery and no patients needed a relook procedure for haemostasis. All patients were seen in the clinic 7 days after surgery for routine endoscopic examination and nasal debridement. They had post-operative magnetic resonance imaging 3 months post-surgery and after 6 months; yearly follow up was planned for all patients.

## Results

The mean volume of blood lost from the 10 patients was 1530 mL (500–4000 mL). The mean volume of re-infused blood was 875 mL (200–2600 mL) (Table 1).

Bacterial growth was detected on all nasal swabs. Organisms that were cultured included: *Staphylococcus aureus, Staphylococcus epidermidis, Streptococcus viridans,* and *Moreaxella catarrhalis* (Table 1).

In both cases in which a LDF was not available, the same organism that was isolated from the nasal cavity was also cultured from the cell saver washed blood. Postoperatively the 2 patients developed a transient bacteraemia with a raised temperature and pulse rate and a positive blood culture, in which the same organism was grown (Table 1). Both were treated with the appropriate intravenous antibiotics according to sensitivities and both recovered completely.

Routine postoperative monitoring revealed that haemoglobin and coagulation levels were within the normal range for all patients, with no evidence of major complications such as haemoglobinuria, coagulopathy, cardiopulmonary issues or sepsis. None of the patients had postoperative epistaxis. None of the patients required any allogeneic blood products. Regular follow up after discharge showed no adverse events (minimum follow-up time was 18 months).

## Discussion and conclusions

The most common benign vascular neoplasm of the nasopharynx is juvenile angiofibroma (JNA) [5]. It is locally destructive and causes bony erosion despite its benign histology. JNA can extend intracranially and cause complications that are potentially life threatening, such as massive blood loss and fatal epistaxis [5]. There are both fibrotic and vascular elements within the tumours, but the vessels lack an elastic lamina and this precludes constriction and is partially responsible for their propensity to bleed [6, 8]. The management of JNA can be challenging because of its aggressive growth pattern, vital adjacent anatomical structures and rich blood supply.

Our institute uses two methods to control blood loss during JNA surgery: the first method is preoperative embolization which was performed in all our patients. The second method is intraoperative cell salvage technique.

Our greatest concern was that we were re-infusing blood from a bacterially colonised surgical field. This has been done previously in gastrointestinal surgery for perforated peptic ulcers. They covered the patients with broad spectrum antibiotics perioperatively and the only post-operative complication they had were wound infections in 3 out of 11 patients, which may have been unrelated to the transfusion [9]. This was reinforced by a study of patients undergoing liver transplantation who received ICS blood transfusion and despite there being positive bacterial cultures in 8 out of 28 re-transfusion bags, no post-operative infections were observed. The patients’ blood cultures remained negative 1 week post-surgery [10]. Other surgical disciplines have also demonstrated the successful use of culture-positive cell saver blood with no reported adverse clinical consequences [11-13]. Another concern associated with cell salvage blood is the promotion of coagulopathy, but no evidence of coagulopathy was revealed in any of our patients, and all laboratory parameters were normal. 

The use of cell salvage autologous blood transfusion is cost effective and reduces the need for allogenic transfusions. This is associated with shorter hospital stays, reduced blood transfusion reactions and reduced postoperative infection rates. The cost of leukocyte filters should be a consideration, especially in resource limited settings. Although these filters are not expensive; additional savings may be instituted by employing a standby technique in JNA surgery, which includes collecting blood from the operative field but only using it if necessary.

The combination of washing blood cells and using a leucocyte filter significantly reduced the bacterial load in processed blood that could then be safely re-transfused to the patient. Leukocyte depletion filters have been widely used during the processing of donated blood to remove white blood cells and their use is now acknowledged to improve cell salvage safety and to reduce the incidence of any cell salvage adverse effects [14]. Various findings have revealed that leukocyte depletion filters are effective in removing white blood cells, tumour cells [15–17], amniotic fluid [18], and bacteria [3].

Our study has shown that cell saver techniques are beneficial in JNA surgery, as the surgery can be completed in a single stage, there is a decreased need for allogenic blood transfusion, and it is often acceptable to Jehovah’s Witness patients. This study has also demonstrated that intraoperative cell salvage is a safe technique in endoscopic JNA surgery. We recommend the use of leucocyte depletion filters and perioperative broad-spectrum antibiotics. Our findings indicate that the commensal bacteria that are introduced with the re-transfused blood are successfully eliminated by an intact immunological system. This explains the good tolerance to the iatrogenic bacteraemia that was caused.

In conclusion, cell salvage autologous blood transfusion is safe and effective in reducing allogenic blood transfusion requirements in endoscopic JNA surgery. Cell salvage should be considered in all cases of JNA surgery as significant blood loss is usually anticipated. This can also be used in situations when patients refuse allogenic blood products. The standby technique allows cell salvage to be used in cases where blood transfusion is not required, but significant bleeding is a possibility, which contributes to relieving the stress on blood donation services. Leukocyte depletion filters are recommended to provide an additional element of safety, and should be used in all cell salvage autologous blood transfusions. In addition, a contaminated surgical field is not a contraindication for the use of cell salvage blood as long as adequate precautions are taken. Recent evidence has shown that cell salvage may be used in malignancy surgery, and that the only contraindication to using cell salvage blood, is the patient’s refusal to accept autologous blood.

A limitation of our study is the small number of patients due to the rarity of the disease. Further research is needed with a larger population sample to define the efficacy of this technique in avoiding allogeneic blood transfusions and its impact on several outcome variables.

## Consent

Written informed consent was obtained from the patients and their parents for publication of this case report and accompanying images. A copy of the written consent is available for review by the Editor-in-Chief of this journal.
